# Synthesis of acute phase proteins in rats with cirrhosis exposed to lipopolysaccharide

**DOI:** 10.1186/1476-5926-5-3

**Published:** 2006-09-12

**Authors:** Susanne Schouw Nielsen, Thorbjørn Grøfte, Niels Tygstrup, Hendrik Vilstrup

**Affiliations:** 1 Department of Medicine V (Hepatology & Gastroenterology), Aarhus University Hospital, Noerrebrogade 44, DK-8000 Aarhus C, Denmark; 2 Department of Medicine A, State University Hospital, 2100 Copenhagen, Denmark

## Abstract

**Background:**

In patients with cirrhosis, infection is frequent and a leading cause of death. This is secondary to various immunologic abnormalities in both the innate and the adaptive immune system. However, it remains unclear whether cirrhosis affects the inflammatory systemic component of the innate immunity, 'the acute phase response', mostly effectuated by the liver itself. We hypothesized that rats with cirrhosis raise a reduced acute phase response induced by lipopolysaccharide (LPS).

**Results:**

We examined the acute phase response induced by intraperitoneal injection of a low dose of LPS, in sham operated control animals and in rats with liver cirrhosis induced by bile duct ligation (BDL). We measured the serum concentrations of the most important acute phase proteins and their liver tissue gene expressions, assessed by mRNA levels. The BDL-model itself increased the serum concentration of α1-acid glycoprotein (α1AGP) and haptoglobin. LPS was lethal to 25% of the cirrhotic animals and to none of the controls. Twenty-four hours after LPS, the serum concentration of α1AGP and haptoglobin, the mRNA level of these acute phase proteins and of α2-macroglobulin and thiostatin rose to the same level in the animals with cirrhosis and in controls.

**Conclusion:**

In rats with experimental cirrhosis LPS caused high mortality. In the survivors, the cirrhotic liver still synthesized acute phase proteins as the normal liver, indicating a normal hepatic contribution to this part of the acute phase response.

## Background

Liver cirrhosis is associated with a high frequency of bacterial infections that increases mortality [1]. The first year after being diagnosed with cirrhosis, patients suffer a more than 40-fold increased mortality from infection compared with the adjusted background population [2]. This reflects multiple immunologic abnormalities secondary to cirrhosis. Attention has focused particularly on the innate immune system, the many protein components of which are synthesized by the liver itself [3]. Thus, it is a frequently held notion that the acute phase response is compromised in cirrhosis patients. However, studies on this subject are few [4,5] and it has not been yet systematically examined in either cirrhosis patients or in experimental models.

The acute phase response consists of changes in the serum concentration of multiple proteins due to a reorganization of hepatic protein synthesis favouring "the acute phase proteins" and decreasing the so-called "negative acute phase proteins". This is part of the non-specific first line of defense against microbes and plays a critical role in the host defense mechanisms, promoting the clearance of invading particles and modulating the immune response against them [6,7].

The proteins acting as acute phase proteins differ from humans to animals and from one species to another. In rat, α1-acid glycoprotein (α1AGP), α2-macroglobulin (α2M), haptoglobin and thiostatin [6,8] are among the major acute phase proteins, whereas C-reactive protein, predominant in humans, does not partake [9]. Albumin reacts as a negative acute phase protein. The acute phase changes in those proteins and their corresponding mRNAs in liver tissue are well described in rats [8,9].

Injection of lipopolysaccharide (LPS), an endotoxin from bacterial cell walls, is a standardized method for induction of the acute phase response. Earlier studies report markedly increased mortality after administration of LPS in rats with experimental liver cirrhosis [10].

We hypothesized that rats with cirrhosis raise a reduced acute phase response induced by LPS. Therefore, we measured the plasma concentrations of selected important acute phase proteins and the expression in liver tissue of their genes assessed by mRNAs. LPS was given four weeks after bile duct ligation (BDL), causing cirrhosis to develop, and 24 hours before examination.

## Results

### Animal and model characteristics

After LPS mortality reached 25% (3/12) among the animals with cirrhosis and there was no mortality in the three other study groups (P = 0.001, Fisher's exact test). No interaction was found between LPS and cirrhosis for either bilirubin, ASAT, liver weight or spleen weight, whereas all of them were increased by cirrhosis (two-way (2-way) ANOVA, P < 0.001). LPS also increased serum bilirubin concentration (2-way ANOVA, P < 0.001) (Table 1).

**Table 1:** Animal and model characteristics

	Bilirubin μmol/l **	ASAT U/l *	Liver weight (gram) *	Spleen weight (gram)*
**Sham**	2.7 (0.5)	332 (143)	9.1 (0.8)	0.64 (0.10)
**Sham+LPS**	3.2 (0.89	345 (156)	8.8 (0.8)	0.76 (0.12)
**Cirrhosis**	39.5 (3.8)	519 (210)	18.5 (2.8)	1.37 (0.42)
**Cirrhosis+LPS**	54.2 (7.8)	912 (406)	16.5 (2.4)	1.27 (0.20)

Values are given as: mean (SD). Bilirubin and ASAT (n = 9) and liver/spleen-weights (n = 10) in sham-operated, in sham-operated injected with LPS (bilirubin and ASAT: n = 10, liver/spleen-weights: n = 12), in cirrhotic (bilirubin and ASAT: n = 11, liver/spleen-weights: n = 12) and in cirrhotic animals injected with LPS (n = 9). **Significantly increased (2-way ANOVA, P < 0.001) by both factors *i.e*. LPS and cirrhosis. *Significantly increased only by cirrhosis (2-way ANOVA, P < 0.001).

### Serum α1AGP, haptoglobin and albumin

**Figure 1:**
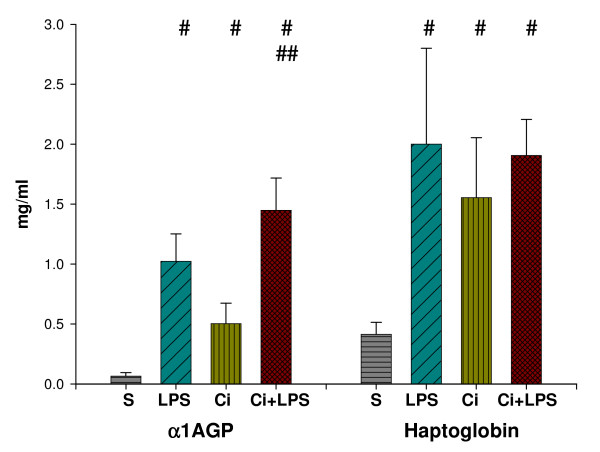
**Serum α1AGP and haptoglobin**. Serum α1-acid glycoprotein (α1AGP) and haptoglobin (mg/ml) in sham-operated (S) (n = 9), in sham-operated injected with LPS (LPS) (n = 10), in cirrhotic (Ci) (n = 11) and in cirrhotic animals injected with LPS (Ci+LPS) (n = 9). Bars represent mean + SD. #Significant difference (1-way ANOVA, P < 0.05) compared with S. ## Significant difference (1-way ANOVA, P < 0.05) for Ci+LPS compared with Ci.

For both α1AGP and haptoglobin, the interaction found between LPS and cirrhosis (2-way ANOVA, P < 0.001) decreased their serum concentrations. The serum concentration – mean (SD) – of α1AGP [S: 0.07 (0.03), LPS: 1.0 (0.23), Ci: 0.5 (0.17), Ci+LPS: 1.5 (0.3) mg/ml] and haptoglobin [S: 0.4 (0.1), LPS: 2.0 (0.8), Ci: 1.6 (0.5), Ci+LPS: 1.9 (0.3) mg/ml] was increased by LPS, cirrhosis and both together compared with the control group (one-way (1-way) ANOVA, P < 0.05). Alpha-1AGP was increased by LPS in the cirrhotic animals (1-way ANOVA, P < 0.05) and haptoglobin tended to increase (Fig. 1).

**Figure 2:**
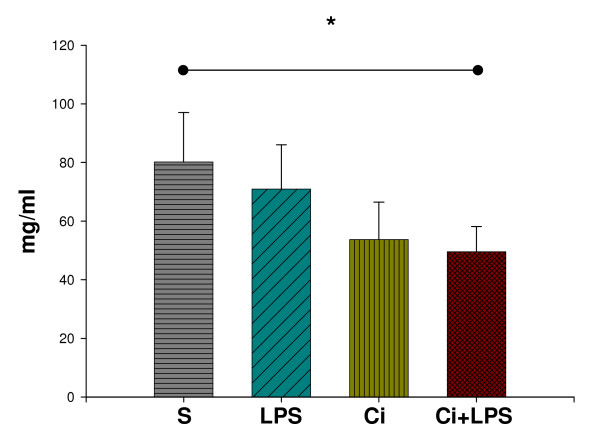
**Serum albumin**. Serum albumin (mg/ml) in sham-operated (S) (n = 9), in sham-operated injected with LPS (LPS) (n = 10), in cirrhotic (Ci) (n = 11) and in cirrhotic animals injected with LPS (Ci+LPS) (n = 9). Bars represent mean + SD. *Significantly decreased by cirrhosis (2-way ANOVA, P < 0.001).

No interaction was found between the factors for serum albumin. Serum albumin [S: 80.2 (16.8), LPS: 70.9 (15.1), Ci: 53.7 (12.8), Ci+LPS: 49.6 (8.6) mg/ml] decreased by cirrhosis (2-way ANOVA, P < 0.001) and not by LPS (Fig. 2).

### Relative mRNAs of acute phase protein genes

In the three treatment groups, the mRNAs were expressed as percentage of mean values of sham-operated animals. There was no interaction between LPS and cirrhosis for any of the mRNAs of the four acute phase proteins: α1AGP [S: 100 (46), LPS: 334 (83), Ci: 166 (41), Ci+LPS: 366 (80)%], haptoglobin [S: 100 (28), LPS: 158 (27), Ci: 144 (20), Ci+LPS: 174 (25)%), α2M (S: 100 (25), LPS: 337 (124), Ci:83 (16), Ci+LPS: 284 (116)%] and thiostatin [S: 100 (24), LPS: 146 (25), Ci: 107 (15), Ci+LPS: 146 (24)%]. The mRNAs of these proteins were increased by LPS (2-way ANOVA, P < 0.001) and only the mRNA of haptoglobin increased also by cirrhosis (2-way ANOVA, P < 0.001).

**Figure 3:**
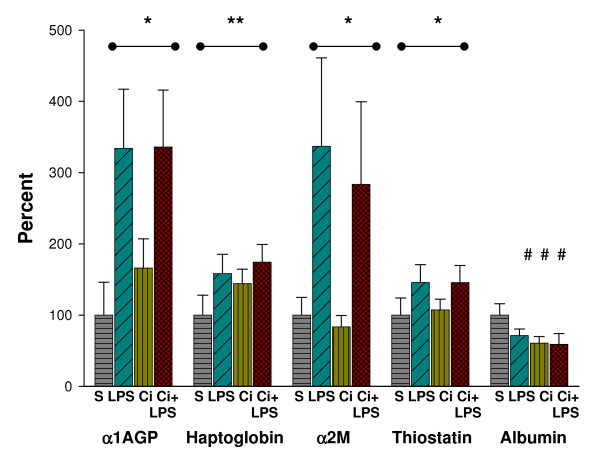
**Relative mRNAs of acute phase protein genes**. Relative mRNA levels for α1-acid glycoprotein (α1AGP), haptoglobin, α2-macroglobulin (α2M), thiostatin and albumin in liver tissue, in sham-operated (S) (n = 10), in sham-operated injected with LPS (LPS) (n = 12), in cirrhotic (Ci) (n = 12) and in cirrhotic animals injected with LPS (Ci+LPS) (n = 9). Bars represent mean + SD, expressed as percentage of mean values of sham-operated animals. **,* Analysed by 2-way ANOVA: **Significantly increased (P < 0.001) by both factors *i.e*. LPS and cirrhosis. *Significantly increased only by LPS (P < 0.001). #Analysed by 1-way ANOVA: #Significant difference (P < 0.05) compared with sham-operated.

There was interaction between the two factors for the mRNAs of albumin that increased the mRNA level. Compared with the control group, the mRNA level of albumin [S: 100 (16), LPS: 71 (9), Ci: 61 (9), Ci+LPS: 59 (15)%] was decreased by LPS, cirrhosis and both together (1-way ANOVA, P < 0.05), and did not change by LPS in the cirrhotic animals (Fig. 3).

### Relation between mRNAs and serum proteins

**Figure 4:**
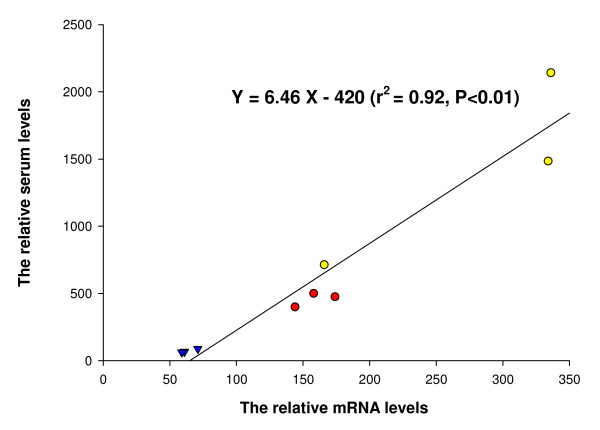
**Relation between relative mRNAs and relative serum concentrations of acute phase proteins**. Correlation between the mean values of the relative mRNA levels and the mean values of the relative serum levels of α1AGP (yellow circles), haptoglobin (red circles) and albumin (blue triangles) in the sham-operated animals injected with LPS (serum levels: n = 10, mRNA levels n = 12), in the cirrhotic (serum levels: n = 11, mRNA levels n = 12) and in the cirrhotic animals injected with LPS (n = 9).

There was a close relationship between the mean values of the relative values of liver tissue mRNAs and mean values of the relative serum levels of α1AGP, haptoglobin and albumin in the three treatment groups (r^2 ^= 0.92, P < 0.01) (Fig. 4).

## Discussion

The aim of our work was to study selected aspects of the acute phase response in an animal model of cirrhosis. The main results were that, in the rats with cirrhosis, LPS caused high mortality and increased serum of α1AGP and of haptoglobin, and also of mRNAs of acute phase proteins to a level as found in the control animals. Furthermore, that the BDL cirrhosis model itself triggered the synthesis of α1AGP and haptoglobin.

Induction of experimental cirrhosis by BDL is a well-described method [11]. The procedure led to cirrhosis (in all animals) with portal hypertension, as indicated by the markedly increased spleen weight [12]. The decreased serum albumin, the increased bilirubin and ASAT by BDL cirrhosis all confirm impaired liver function. Nonetheless, the increased liver weight by the model may reflect some extent of maintained hepatocellular mass [13]. One of our recent and not yet published studies has shown that the BDL model does not seem associated with loss of hepatic functional reserve in terms of reduced galactose elimination capacity (GEC). Therefore, the model is probably not one of clinical end-stage liver disease.

Activation of the innate immune system by LPS is well known and LPS treatment is one of the most commonly used methods for inducing the acute phase response. Rats with BDL are highly sensitive to LPS [10] – one study reported increased mortality after down to 0.01 mg/kg LPS. We chose to use a dose of 0.5 mg/kg LPS, which is reported to markedly increase mortality [10]. The mortality of 25% in our animals with cirrhosis and no mortality in the controls matches other reports [10,14].

The acute phase proteins are playing different roles in the acute phase response. Some initiate or sustain the response, others have tissue-protective or anti-inflammatory actions [7]. The four proteins determined in this study are among the best indicators of the acute phase response in the rat [6,8]. The presented study includes an estimate of their gene expression and also the serum concentration of α1AGP and haptoglobin. The serum concentrations of α2M and thiostatin were not obtained, as the analyses for these proteins are not commercially available. The role of α1AGP is not clear; but it seems to have anti-inflammatory functions [15]. Haptoglobin conserves iron released from haemoglobin [9]. Alpha-2M and thiostatin are plasma proteinase inhibitors protecting against proteolytic auto-degradation [6,16].

The decrease of certain proteins during the acute phase response is presumably caused by the need to divert available amino acids into the production of active acute phase proteins. Albumin is one of these proteins. The lack of a significant reduction of albumin by the dose LPS used in this study corresponds to other reports [10] and indicates induction of a mild acute phase response.

We found a close and linear relation between the relative values of mRNAs and relative serum levels of α1AGP, haptoglobin and albumin in the three treatment groups. This result suggests that the changes in serum of those proteins by the acute phase response, by cirrhosis, and by both together, were determined to a large extent by changes in expression of their genes although we have no data on posttranslational events. This is in line with earlier studies on rats without cirrhosis [17].

The increase of α1AGP and haptoglobin in serum as well as of the mRNA of the later by BDL shows that this cirrhosis model in itself induced an acute phase response, probably because of the active fibrogenesis acting as an inflammatory process. Conversely, those proteins may themselves have a modulating effect on the fibrotic process [18,19]. There are several reports indicating increased acute phase protein synthesis also in human cirrhosis [5,20].

Our data show that during the acute phase response, the cirrhotic liver still synthesized haptoglobin and α1AGP and, probably, also α2M and thiostatin, as normal livers do. This is line with our earlier findings, that the synthesis of the acute phase proteins benefits from high metabolic priority during decreased functional liver mass caused by high dose LPS treatment [21] and by hepatectomy [22].

We found an interaction between the effects of LPS and cirrhosis on the mRNAs of albumin that increased the mRNA level. However, this trend was not present for the serum concentration of the protein. Moreover, a decreasing interaction was found between the two factors on the serum concentration of both α1AGP and haptoglobin; thus, both these two proteins increased less by LPS in the cirrhotic than in the non-cirrhotic animals. Further information on whether this just reflects that the synthesis of those proteins already is increased by the BDL model itself or reflects suppression of the acute phase response by cirrhosis is not provided in this study. The sufficiency of the response seems, however, more likely to be reflected by the concentrations of the acute phase proteins during the response, rather than their exact increase. If so, our study rejects the idea that the markedly increased mortality of patients with cirrhosis exposed to infection is rooted in a decreased liver function in the form of a decreased synthesis of acute phase proteins. In addition, that the function of the phylogenetically old, innate immune system is robust during cirrhosis, and that alternative explanations to the clinical immune deficiency should be sought for.

The synthesis of the acute phase proteins, during the response, is considered to constitute a high part of total body protein synthesis and to be highly demanding in metabolic terms [23-25]. We speculate that the maintenance of this protein synthesis by the cirrhotic liver may happen at the expense of other metabolic processes. One of our recent not yet published studies indicates that this is the case regarding the capacity of urea synthesis. With the reservation that the presented data were obtained from the group of survivors, we furthermore speculate that the high mortality of the LPS-exposed cirrhotic rats was related to the metabolic demanding synthesis of the acute phase proteins.

Limitations in the interpretation of the presented data are that the animals with cirrhosis already exhibited increased production of α1AGP and haptoglobin and that the data were obtained only from the group of survivors. In addition, defining the acute phase response by the indicated proteins is a narrowly defined approach, as the response includes many physiological reactions.

## Conclusion

Low dose LPS caused markedly increased mortality in rats with experimental cirrhosis. In the survivors, the synthesis of the acute phase proteins remained intact, indicating a normal hepatic contribution to this part of the acute phase response. We speculate that the increased sensitivity to LPS in the cirrhotic animals may be related to the metabolically demanding acute phase protein synthesis. The data should, however, be interpreted with caution and further studies on other cirrhosis models are required.

## Materials and methods

### Animals

Female Wistar rats (body weight 200–210 g; Taconic, Ejby, Denmark) were housed at 19 ± 3°C, with a 12-hours (06:00 AM–06:00 PM) artificial light cycle, with two or three animals from the same treatment group per cage. They had access to tap water and standard food (Altromin, Lage, Germany) *ad libitum*, during the whole experiment. The study was undertaken in accordance with prevailing local and national guidelines for animal welfare and approved by the Experimental Animal Inspectorate.

### Design

We studied four groups: two groups of sham-operated animals injected with NaCl or LPS, and two groups of BDL animals, injected with NaCl or with LPS 24 hours before experimental examination: 1) Sham-operated animals injected with NaCl (S); 2) Sham-operated animals injected with LPS (LPS); 3) BDL-operated animals injected with NaCl (Ci); 4) BDL-operated animals injected with LPS (Ci + LPS).

Bilirubin, ASAT, α1AGP, haptoglobin and albumin were determined in the sham-operated (n = 9), in the sham-operated injected with LPS (n = 10), in the BDL-operated (cirrhotic) (n = 11) and in the BDL-operated (cirrhotic) animals injected with LPS (n = 9). The mRNAs and the liver and spleen weights were determined in the sham-operated (n = 10), in the sham-operated injected with LPS (n = 12), in the BDL-operated (cirrhotic) (n = 12) and in the BDL-operated (cirrhotic) animals injected with LPS (n = 9).

### BDL, sham-operation and acute phase response induction

BDL and sham-operation was performed under anaesthesia with 0.5 ml/kg Hypnorm s.c. (fentanyl/fluanisone; Jansen Pharma, Birkeroed, Denmark) and 0.5 ml/kg Dormicum (5 mg/ml) s.c. (midazolam; La Roche, Basel, Schwitzerland). Following a midline abdominal incision, the common bile duct was isolated, triple ligated with 3-0 monofil polyamid and sectioned between the ligatures. The sham operation consisted of isolation and gentle manipulation of the common bile duct.

Twenty-five to 30 days after operation, the animals were injected intraperitoneally with either 0.5 mg/kg LPS (from *Ecsherichia coli *obtained from Sigma (0111:B4) (catalogue no. L2630) Vallensbaek, Denmark) dissolved in 0.5 ml isotonic NaCl or the vehicle.

### Cirrhosis determination

After sacrifice, the spleen and liver were weighed. Liver tissue from all BDL-operated animals was fixed overnight in formalin, embedded in paraffin and stained with hematoxylin-eosin and Masson's trichrome, for histological examination. Classification as cirrhotic required macroscopic cirrhosis (micro-nodular surface) and microscopic diffuse architectural changes, with proliferation of bile duct-like structures with fibrosis and solid porto-portal septa formation. These criteria were satisfied in all BDL-operated animals.

### Serum acute phase proteins, bilirubin and aspartate aminotransferase

Alfa-1AGP, haptoglobin and albumin concentrations in serum were determined using an ELISA kit specific for the rat proteins (Alpha Diagnostic, San Antonio, Texas and Life Diagnostics, West Chester, UK) based on the manufacturer's instructions. Samples were assayed in duplicate. The lower limit of detection was 1.56 ng/ml for α1AGP, 0.98 ng/ml for haptoglobin and 50 ng/ml for albumin. Analyses for α2M and thiostatin are not commercially available. Serum albumin and haptoglobin were also measured by clinical routine analytical methods (Bromcresol Green and Immune Turbidimetric analysis) and results correlated closely with those obtained by the ELISA kits. Bilirubin and aspartate aminotransferase (ASAT) were determined by routine analytical methods.

### mRNAs

Following anaesthesia as used for BDL and sham operation (cf. above), about 200 mg of liver tissue from the left lobe was snap-frozen in liquid N and stored at -80°C. The mRNA levels of the rat acute phase proteins α1AGP, α2M, thiostatin, haptoglobin and albumin were semi-quantified.

Total RNA was isolated with RNeasy^® ^Midi Kit (Quiagen, Hilden, Germany) and mRNA levels were measured by slot blot hybridisation as previously described in detail [26]. Hybridization was performed with QuickHyb^® ^hybridisation solution (Stratagene, La Jolla, California) at 68°C for 1 hour, followed by stringent wash. The intensities of the hybridisation signals were quantified by phosphorimaging with a Fujix Bioimaging Analyzer System BAS2000 (Fuji Photo Film, Tokyo, Japan). After visualisation of the radioactive signal, the blots were analysed with Tina Version 2.09c software (Ray Test, Fuji Photo Film, Tokyo, Japan) and the results were expressed as photostimulated luminescence (PSL) units corrected for background per unit area (PSL/S, *i.e*., [PSL-background]/mm^2^). Values were expressed as percentage of the mean value of the control animals. No pools were made and each animal was a unique value.

The cDNA probes were built according to published data, as follows: α1AGP [27]; α2M [28]; thiostatin [29]; haptoglobin [30]; albumin [31]. The DNA fragments were separated by agar gel electrophoresis and eluted on Spin Bind DNA Extraction Units (FMC).

### Statistical methods

Statistical analyses were performed with the SPSS (version 11.0; SPSS Inc., Chicago, IL). All data are presented as means (SD). Data was tested for normal distribution by Q-Q plot in each study group and homogeneity of variance assumption by Bartlett's test. In order to establish homogeneity of variances, the results of serum α1AGP and haptoglobin, the mRNA of α2M, the ASAT, the bilirubin and the liver and spleen weights were logarithmic transformed. Data were analysed with two-way (2-way) ANOVA. In case of interaction between the factors, the data were analysed with one-way (1-way) ANOVA. Correction for multiple testing was performed with Bonferroni. Mortality was analysed with Fisher's exact test and associations with Pearsons's correlation.

## Competing interests

The author(s) declare that they have no competing interest.

## Authors' contributions

SSN conceived the design of the study and carried out the experiments. TG participated in conceiving the design of the study. NT performed the mRNA analysis. HV and SSN drafted the paper. All authors read and approved the paper.
